# The photosensor protein Ppr of *Rhodocista centenaria *is linked to the chemotaxis signalling pathway

**DOI:** 10.1186/1471-2180-10-281

**Published:** 2010-11-09

**Authors:** Sven Kreutel, Andreas Kuhn, Dorothee Kiefer

**Affiliations:** 1 Institute of Microbiology and Molecular Biology, University of Hohenheim, Garbenstrasse 30 D-70593 Stuttgart, Germany

## Abstract

**Background:**

*Rhodocista centenaria *is a phototrophic α-proteobacterium exhibiting a phototactic behaviour visible as colony movement on agar plates directed to red light. As many phototrophic purple bacteria *R. centenaria *possesses a soluble photoactive yellow protein (Pyp). It exists as a long fusion protein, designated Ppr, consisting of three domains, the Pyp domain, a putative bilin binding domain (Bbd) and a histidine kinase domain (Pph). The Ppr protein is involved in the regulation of polyketide synthesis but it is still unclear, how this is connected to phototaxis and chemotaxis.

**Results:**

To elucidate the possible role of Ppr and Pph in the chemotactic network we studied the interaction with chemotactic proteins *in vitro *as well as *in vivo*. Matrix-assisted coelution experiments were performed to study the possible communication of the different putative binding partners. The kinase domain of the Ppr protein was found to interact with the chemotactic linker protein CheW. The formation of this complex was clearly ATP-dependent. Further results indicated that the Pph histidine kinase domain and CheW may form a complex with the chemotactic kinase CheAY suggesting a role of Ppr in the chemotaxis signalling pathway. In addition, when Ppr or Pph were expressed in *Escherichia coli*, the chemotactic response of the cells was dramatically affected.

**Conclusions:**

The Ppr protein of *Rhodocista centenaria *directly interacts with the chemotactic protein CheW. This suggests a role of the Ppr protein in the regulation of the chemotactic response in addition to its role in chalcone synthesis.

## Background

*Rhodocista centenaria*, first described as *Rhodospirillum centenum *[1] is a thermotolerant phototrophic purple bacterium of the α-proteobacteria group isolated from hot springs in Wyoming 1985. The slightly spiroid or vibrioid shaped cells are motile by means of a single long flagellum, their intracellular photosynthetic membranes are lamellar and their *in vivo *absorption spectra show features almost indistinguishable from those of *Rhodospirillum rubrum *[2]. However, 16S rRNA analysis elucidated considerable differences between the species, hence *Rhodocista *was separated into a new genus [3], now consisting of three species [4,5]. *R. centenaria *is closely related to the plant-associated genus *Azospirillum *[6]. As virtually all phototrophic organisms, *R. centenaria *exhibits a sensory response to light originally described as "Schreckbewegung" [7]. Engelmann and also Manten [8] found that *R. rubrum *cells accumulated in the most intense area of light gradients between wavelengths 800 and 900 nm. *R. centenaria *shows a particularly unique form of macroscopic phototactic behaviour, first described in 1994 by Gest and coworkers [9]. On solid media, the phototactic colonies move towards longwave light and away from light with wavelengths less than 650 nm [10]. *R. centenaria *develops lateral flagella in viscous media or on solidified surfaces. These flagella consist of a distinct flagellin whose expression is controlled by specific *mot *and *fli *genes [11].

For *R. centenaria*, a close relationship between chemotaxis and the phototactic response has been found [12]. As seen with many other photosynthetic bacteria, *R. centenaria *has multiple chemotaxis operons with distinct functions [13-15]. The chemotaxis gene cluster has been well characterized and most of the genes are similar to those of other Gram negative bacteria like *Escherichia coli*. In brief, the histidine kinase CheA is linked to the chemotactic receptors (MCPs) by the CheW protein [16]. This trimeric receptor complex controls the phosphorylation level of the response regulator CheY. Activated CheY (CheY-P) interacts directly with the motor of the flagella to control swimming direction. The dephosphorylation of CheY-P occurs spontaneously, only in enterobacteria this reaction is accelerated by the phosphatase CheZ. For adaptation, CheB and its antagonist CheR remove or add methyl groups to the receptors, respectively.

In *R. centenaria*, the two central components of the chemotactic signal transduction cascade, namely CheA and CheY, are present as the fusion protein Rc-CheAY located in the first chemotactic operon [17], a situation that is also observed in *Helicobacter *[18]. Whereas the role of the CheY-domain of the CheAY protein in *H. pylori *seems to be a phosphate sink, in *R. centenaria*, the function of Rc-CheAY remains still unclear. While Che proteins are generally involved in chemotactic responses, they were also shown to affect the phototactic response in *R. centenaria *as demonstrated by the analysis of many *che *mutants [19].

In the last decade, bacterial photoreactive proteins like phytochromes, previously thought to be a unique feature in plants, have been identified as photoactive yellow proteins (Pyp) and have now been extensively studied in a variety of eubacterial species (for review see [20,21]). For *R. centenaria*, a Pyp-like protein, Ppr, was described in 1999 by Bauer and colleagues [22]. The large fusion protein Ppr consists of three functional domains, an N-terminal Pyp domain with the cinnamic acid chromophore, the central phytochrome-like bilin attachment domain Bbd and the C-terminal histidine kinase domain Pph which autophosphorylates an essential histidine residue [22]. Although some Pyp proteins have been crystallized and biophysically characterized in great detail (reviewed by [21]), no distinct physiological role could be attested to these unique proteins. A Ppr-deletion mutant lacking amino acid residues 114-750 did not show any alterations in phototactic behaviour, instead exhibited a strongly deregulated expression of the chalcone synthase gene suggesting a regulatory function in the polyketide synthesis [22]. Although there is no obvious direct involvement of Ppr in the phototactic or scotophobic reaction, an interaction with the chemotactic signal transduction components is plausible to regulate general phosphorylation levels or transduce phosphoryl groups to a yet unknown light-dependent signal transducing protein. We therefore analysed whether the Ppr protein and in particular its phosphorylating kinase domain Pph interacts with the Rc-Che proteins.

## Results

### The chemotactic response of *E. coli *is inhibited by the expression of Ppr

**Figure 1:**
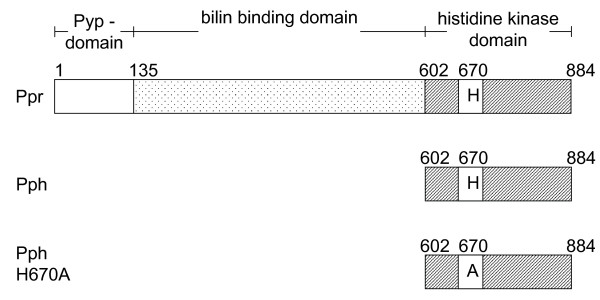
**Domain structure of the Ppr photosensor protein of *R. centenaria*. **The Ppr protein consists of a photoactive yellow protein domain (Pyp; residues 1-135) which carries the blue light absorbing chromophore *p*-hydroxycinnamic acid, a central bacteriophytochrome bilin binding domain (residues 136-601) with the red light absorbing biliverdin chromophore, and a histidine kinase domain (Pph; residues 602-884). The truncated Pph protein consists of the histidine kinase domain (residues 602-884). In the mutant Pph H670A the putative autophosphorylated histidine residue (H670) is replaced by an alanine.

**Figure 2:**
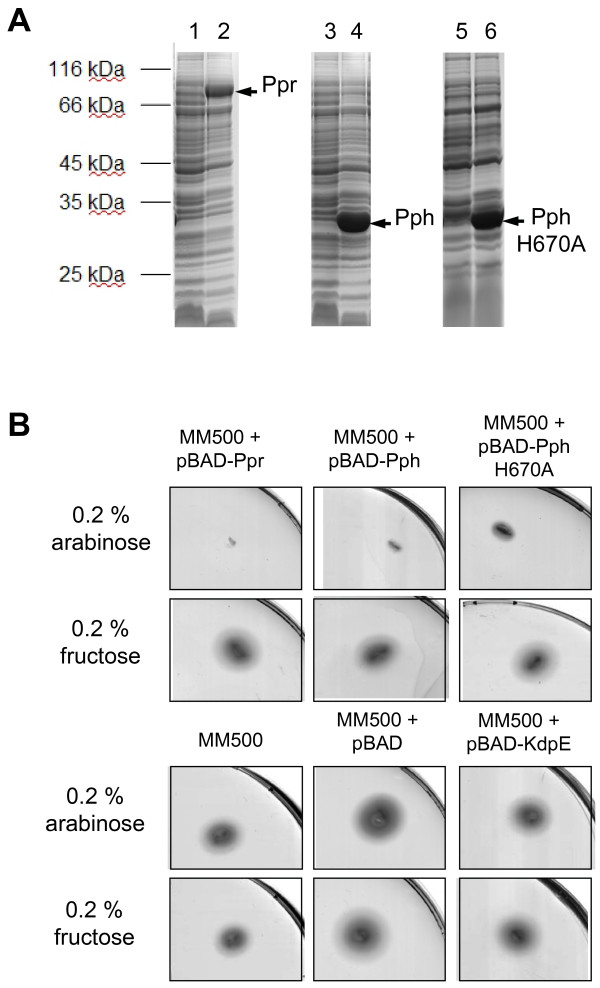
**Chemotaxis of *E. coli *is inhibited by the expression of Ppr or Pph. **(A) The chemotactic wild type strain *E. coli *MM500 was transformed with the plasmids pBAD-Ppr (lanes 1 and 2), pBAD-Pph (lanes 3 and 4) and pBAD-Pph H670A (lanes 5 and 6). Cells were grown in TB medium to an OD_600 _= 0.5, 0.2% fructose (lanes 1, 3 and 5) or 0.2% arabinose (lanes 2, 4 and 6) was added, and growth was continued for 3 hours. Protein expression was analyzed by SDS-PAGE and Coomassie blue staining. The positions of molecular weight markers are indicated. (B) TB swarm agar plates containing either 0.2% arabinose or 0.2% fructose as indicated were inoculated with the following cells. Upper panels: *E. coli *MM500 transformed with plasmids pBAD-Ppr, pBAD-Pph or pBAD-PphH670A, respectively. Lower panels: Untransformed MM500 cells, MM500 transformed with plasmids pBAD or pBAD-KdpE, respectively. To develop chemotactic rings the plates were incubated for 6 hours at 37°C.

The chemotactic network in *E. coli *is very sensitive to alterations in the expression level and stoichiometry of the chemotactic proteins Ec-CheW [23,24] and Ec-CheA [25] as well as the MCP receptors [26,27]. When high amounts of single proteins of the chemotactic network are expressed a non-responding chemotactic phenotype is observed, similar to a *che *deletion mutant [28]. To investigate whether the Ppr protein of *R. centenaria *participates in the chemotactic network, Ppr and, in particular, its histidine kinase domain Pph were overexpressed in the chemotactic wild-type strain *E. coli *MM500. To this end, the plasmids pBAD-Ppr, pBAD-Pph and pBAD-PphH670A encoding the entire photoreceptor Ppr, the C-terminal histidine kinase domain Pph and the mutant PphH670A protein, respectively (Figure 1), were used to transform *E. coli *MM500. These plasmids carry the cloned genes under the control of the arabinose-inducible araBAD promoter. First, protein expression was analyzed by SDS-PAGE and Coomassie-blue staining. All three Ppr-derived proteins were expressed in the presence of arabinose (Figure 2A, even numbered lanes) but not in the presence of fructose (odd numbered lanes). Next, the chemotactic behaviour of the transformed cells was assayed. TB swarm agar plates, containing either arabinose or fructose were inoculated with the respective cells, incubated for 6 hours at 37°C and the swarm diameters were compared (Figure 2B). The chemotactic response of the wild type strain *E. coli *MM500 without or with the empty pBAD vector was clearly visible by the formation of a swarming ring (lower left and central panels). The response was completely abolished when cells containing the plasmids pBAD-Ppr or pBAD-Pph were grown in the presence of arabinose. In these cases no swarm rings were visible (upper left and central panels). However, the expression of the mutant protein Pph-H670A where the histidine residue at position 670 has been substituted with an alanine residue, led to an only intermediate chemotactic response (upper right panel). The histidine residue at 670 of Pph is a putative phosphorylation site and is located in a H-box region [29]. All strains were also analyzed on swarm plates containing 0.2% fructose that did not induce the expression of the Ppr proteins and did not significantly affect the size of the swarming rings (Figure 2B). As a control, the histidine kinase KdpE from *R. centenaria *was overexpressed which did not interfere with the chemotactic swarming (lower right panel). To rule out that the inhibitory effect on chemotaxis is caused by a reduced growth rate due to the heterologous expression of the *Rhodocista *proteins, growth curves of induced and non-induced and empty plasmid control cells were recorded and compared. No differences in growth rates depending on the presence of arabinose or fructose in the media were found (data not shown).

**Figure 3:**
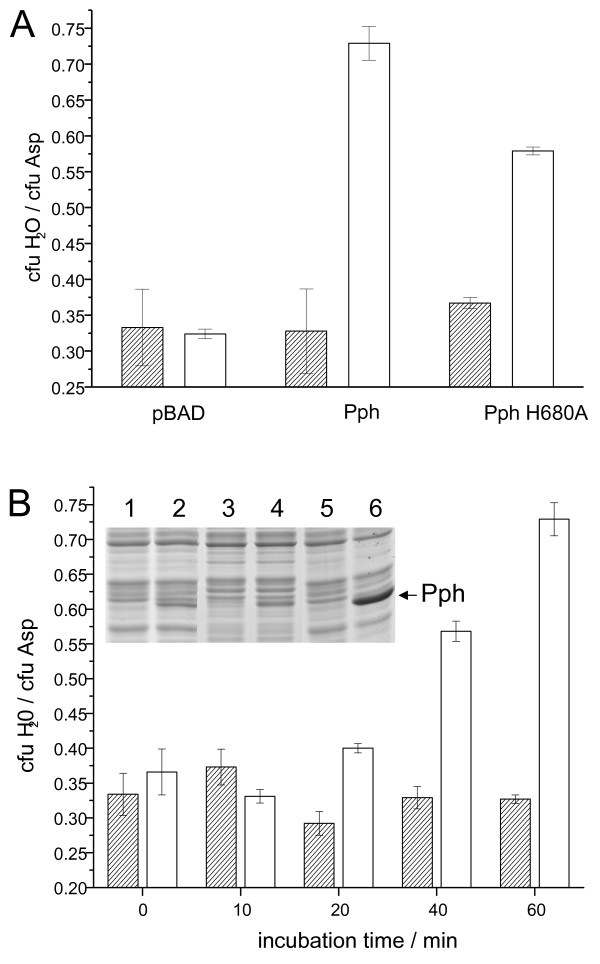
***E. coli *cells expressing the Pph protein are unable to respond to aspartate. **(A) The chemotactic response to aspartate of *E. coli *MM500 cells expressing the various Pph-derived proteins was investigated with a chemotactic chamber. The chemotactic inhibition (CI) was calculated as described in Materials and Methods. The CI-value of cells grown in the presence of fructose (hatched columns) was about 0.35, whereas cells grown in the presence of arabinose and expressing the Pph or the Pph-H670A protein (white columns) were calculated to 0.73 or 0.58, respectively. The error bars indicate the standard deviations of three independent experiments. (B) *E. coli *cells with pBAD-Pph were incubated for the indicated times with 0.2% arabinose or 0.2% fructose, respectively, and their chemotactic response to aspartate was investigated in a chemotactic chamber. The chemotactic inhibition rate was calculated after induction either with fructose (hatched columns) or arabinose (white columns) for the indicated time points. The error bars indicate the standard deviations of three independent experiments. The protein expression profiles (inlet) were analysed at 10 min (lanes 1, 2), 40 min (lanes 3, 4) and 60 min (lanes 5, 6) after induction. The odd numbered lanes are the non-induced controls.

To investigate the inhibitory effect of the Ppr protein on chemotaxis in more detail capillary assays with a chemotactic chamber [30] were performed. *E. coli *MM 500 was transformed with pBAD-Pph and pBAD-PphH670A, respectively. The cells were grown in minimal medium A (MMA) containing 0.2% fructose as a carbon source, and the heterologous protein expression was induced by the addition of arabinose when the culture reached an optical density of 0.6. The number of cells entering a capillary containing the attractant aspartate (1 mM) was determined after 30 min of incubation. To normalize the chemotactic activity the chemotactic inhibition (CI) was evaluated by dividing the colony forming units in the control samples (cfu H_2_O) by the colony forming units in the experiment onset (cfu Asp). Consequently, a high CI value indicates that the chemotactic response is blocked whereas a low CI value reflects a normal chemotaxis. *E. coli *cells expressing Pph showed a nearly complete absence of a chemotactic response to aspartate after 60 min (Figure 3A, central white column). The chemotactic inhibition was calculated to 0.73. In contrast, cells grown with 0.2% fructose (hatched columns) or cells harbouring the pBAD vector (left columns), showed a CI of approximately 0.35. Corroborating the results with the swarm plates shown in Figure 2B, the expression of the Pph-H670A mutant protein lead to an only reduced chemotactic inhibition of 0.58 and did not reach the wild type CI value. To check whether the inhibitory effect depends on the amount of Pph protein, capillary chemotaxis assays with different induction times were performed (Figure 3B). At the respective time, the expression of Pph was analysed by SDS-PAGE (inlet). Our results indicate that the chemotactic inhibition increases with time and depends on the amount of Pph protein expressed. A similar effect of Ec-CheW overexpression on chemotaxis has been observed [26]. The cellular protein level of Pph was verified in parallel by SDS-PAGE and Westernblot analysis (data not shown). Taken together, the results strongly indicate that the Pph interferes with the chemotactic pathway in *E. coli*.

### The Pph protein interacts with Rc-CheW in an ATP-dependent manner

**Figure 4:**
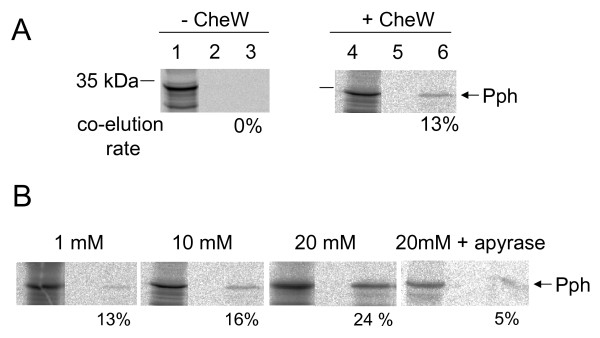
**Interaction between Pph and the chemotactic protein Rc-CheW. **(A) The binding of the histidine kinase domain Pph and CheW was analyzed in pull-down assays. *R. centenaria *6his-Rc-CheW was expressed in *E. coli *C41 cells and purified. The Pph protein was translated *in vitro *in the presence of [^35^S]-methionine (lane 1 and 4). Rc-CheW was added (50 μg) to the reaction and incubated at 37°C. The sample was applied to a Cu-Sepharose column and after washing the bound complexes were eluted (lanes 3 and 6). The fractions were analysed by phosphorimaging. The *in vitro *translating protein extracts are shown in lanes 1 and 4, the final wash steps in lanes 2 and 5 and the elution fractions in lanes 3 and 6, respectively. The co-elution rate was calculated and is indicated. The positions of molecular weight markers are indicated. (B) The binding of the Pph protein and Rc-CheW was analysed in the presence of ATP. The Pph protein was translated and Rc-CheW was added as described in (A). ATP or apyrase was added to each reaction as indicated and the samples were analysed as described in (A). The co-elution rate was calculated and is indicated in % as bound Pph protein.

To investigate in detail with which components of the Rc chemotactic pathway Ppr and its C-terminal histidine kinase domain Pph interact, the binding to Rc-CheW or Rc-CheA was analyzed. First, purified *R. centenaria *CheW (Rc-CheW) containing an N-terminal his-tag and *in vitro *translated and radiolabelled Pph protein were tested for interaction by matrix-assisted coelution. The Rc-CheW protein as a bait was heterologously expressed in *E. coli *C41 and purified by immobilized metal affinity chromatography (Cu-IMAC). The prey protein Pph was translated *in vitro *and labelled with [^35^S]-L-methionine (Figure 4A, lanes 1 and 4). To avoid unspecific binding of Pph to the Cu Sepharose, a buffer containing 50 mM imidazole was used. In the assay, both the bait and prey protein were mixed, incubated overnight at 37°C and then bound to the Cu Sepharose column. After intensive washing the bound protein was eluted, separated by SDS-PAGE and analysed by autoradiography. As shown in Figure 4A, the Pph protein co-elutes in the elution fractions containing Rc-CheW (lane 6) whereas no Pph protein was detected in the elution fraction of the control without Rc-CheW (lane 3). The co-elution rate was calculated to 13% of the input Pph protein (lane 4). To address the possible role of ATP in the binding process of the histidine kinase domain to Rc-CheW, co-elution experiments in the presence of increasing amounts of added ATP (0-20 mM) were performed. The data clearly show that the stepwise addition of ATP increased the amount of the Rc-CheW-bound Pph up to 24% (Figure 4B). When, for a control, the residual ATP was hydrolyzed by adding apyrase, the binding decreased to 5%. It should be considered that in all experiments a low ATP level (2 mM) is required to allow *in vitro *transcription and translation. This explains why in the experiment with apyrase a lower binding was observed than when no additional ATP was added.

**Figure 5:**
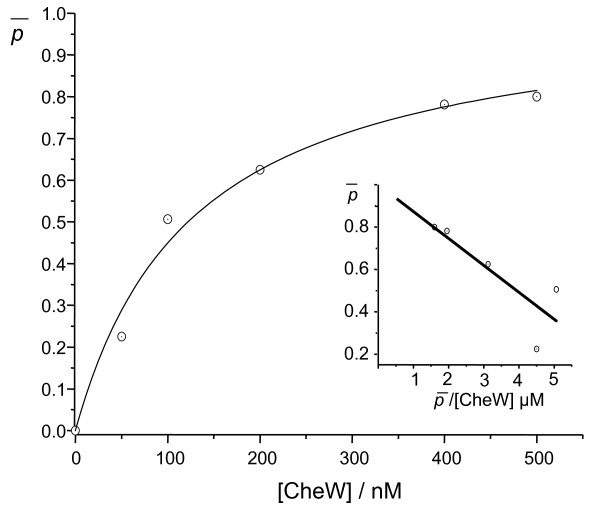
**Binding of the histidine kinase domain Pph to Rc-CheW. **Purified Pph protein was immobilized on an aminosilane cuvette and increasing amounts of Rc-CheW were added. After 30 minutes of incubation the free protein was removed and the bound Rc-CheW was calculated. The corresponding Scatchard plot is shown in the inlet.

To calculate the dissociation constant (K_d_) of the binding between the histidine kinase domain Pph and Rc-CheW, resonant mirror spectroscopy experiments with a biosensor cuvette system were performed. For these experiments Pph with a C-terminal strep-tag and an N-terminal his-tag was purified by immobilized metal affinity chromatography (Cu-IMAC). An aminosilane cuvette was activated and coated with streptactin. The purified Pph protein was then bound via its strep-tag to the immobilized streptactin. Increasing concentrations of purified Rc-CheW were added and the binding was recorded during 30 minutes. The amount of bound Rc-CheW and the fractional saturations ($\bar{p}$) were calculated for each experiment and the data were displayed in a plot against the added Rc-CheW concentration (Figure 5). A hyperbolic binding curve was revealed and the dissociation constant was calculated to K_d _= 0.13 ± 0.03 μM. Therefore, the binding of the histidine kinase domain Pph to Rc-CheW of *R. centenaria *appears to be stronger than the binding between the histidine kinase Ec-CheA and Ec-CheW that has been analysed in *E. coli *[31].

### The histidine kinase Pph is present in a complex with Rc-CheW and Rc-CheAY

**Figure 6:**
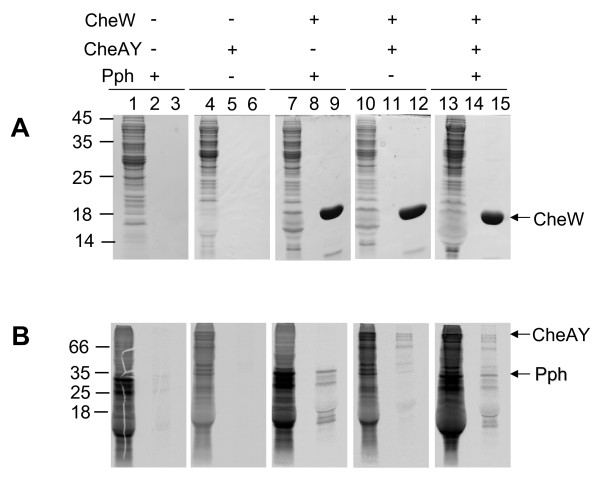
**Interaction of the Pph-CheW complex with Rc-CheAY. ***In vitro *translated [^35^S]methionine radiolabelled Pph and Rc-CheAY proteins were mixed with purified CheW-6his, incubated at 37°C and bound to Cu-Sepharose. After extensive washing the complexes were eluted and the fractions were analysed by SDS-PAGE and Coomassie blue (A) or by autoradiography (B). The proteins added in each experiment are depicted by +. The reactions containing the *in vitro *translated protein in total are shown in lanes 1, 4, 7, 10 and 13. The last washing steps are shown in lanes 2, 5, 8, 11 and 14 and the elution fractions in lanes 3, 6, 9, 12 and 15. The positions of molecular weight markers are indicated.

Since the chemotactic MCP receptor proteins in *E. coli *and *Rhodobacter sphaeroides *were found in heterooligomeric complexes together with CheW and CheA [32-34], we investigated whether the Pph protein can bind to Rc-CheAY in the presence of Rc-CheW. Pull-down experiments with purified Rc-CheW containing an N-terminal his-tag and *in vitro *translated and radioactively labelled Pph and Rc-CheAY proteins were performed (Figure 6). The translation reaction with added Rc-CheW protein was incubated overnight and loaded on an affinity column (Cu Sepharose). Unbound proteins were removed by extensive washing steps and the specificly bound proteins were eluted by imidazol and analyzed by SDS-PAGE, Coomassie staining and autoradiography. The Pph protein as well as Rc-CheAY co-eluted together with Rc-CheW (Figure 6, lanes 15). In addition to the CheAY and Pph protein bands at the expected positions, smaller bands were detected that presumably result from incomplete translation of Pph and Rc-CheAY, respectively. The results indicate that a complex composed of Rc-CheW, CheAY and the histidine kinase domain Pph may be formed *in vitro*. When Rc-CheAY protein was incubated with only Rc-CheW, it was also found in the elution fraction (lane 12) suggesting that Rc-CheAY itself binds to Rc-CheW. This result is not unexpected since in *E. coli *Ec-CheA is also found attached to Ec-CheW (for a recent review see [35]). When only the Pph protein was incubated with Rc-CheW (lane 9), both proteins co-eluted from the Cu Sepharose column, showing that Pph presumably binds directly to Rc-CheW. As control experiments, the proteins were analysed in the absence of Rc-CheW (lanes 3 and 6) showing no elution of Pph or Rc-CheAY.

Taken together, the results give preliminary evidence that the C-terminal histidine kinase domain Pph of the photosensor protein Ppr assembles *in vitro *into a trimeric complex of Pph, Rc-CheW and Rc-CheAY.

### The oligomeric state of the histidine kinase domain Pph

**Figure 7:**
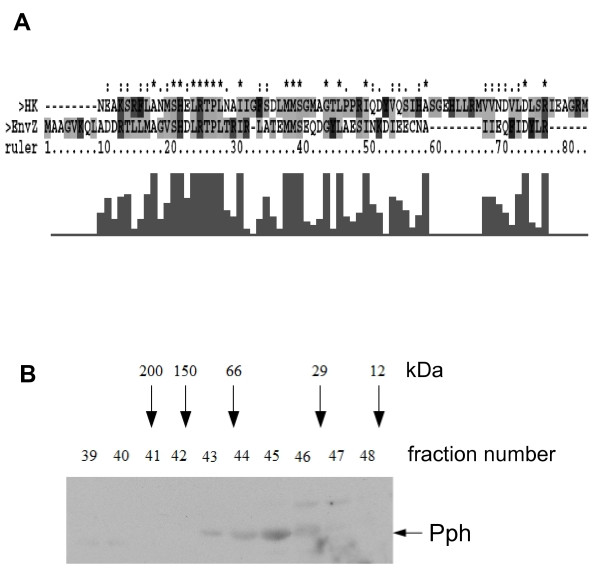
**Oligomeric state of the histidine kinase Pph. **(A) Alignment of the dimerization domains of the Pph protein from *R. centenaria *and EnvZ from *E. coli*. The identity was 27% whereas the similarity was calculated with about 57%. The alignment was performed with the Clustal X software. (B) Purified Pph was analysed by gel filtration on a Sephadex G-200 column. Aliquots of the elution fractions (39-48) were separated by SDS-PAGE and blotted on a nitrocellulose membrane. The Pph protein was detected with a conjugate raised against the C-terminal StrepTag II. The position of the Pph protein is indicated. The following proteins werde used as molecular weight markers: β-amylase (200 kDa), alcohol dehydrogenase (150 kDa), albumin (66 kDa), carboanhydrase (29 kDa) and cytochrome c (12 kDa) were used.

Sequence alignments of the Pph domain with other bacterial histidine kinases (e.g. EnvZ, KdpD and PhoR) identifies a predicted dimerization motif in the N-terminal part of Pph. The Pph sequence shows an identity of 27% and a similarity of about 57% compared to the dimerization domain of EnvZ (Figure 7A). To investigate whether the Pph protein can form a dimer *in vitro*, we performed gel filtration under non-reducing conditions. Crude soluble extracts of Pph expressing *E. coli *cells were separated on a Sephadex G-200 column and analyzed by SDS-PAGE and Westernblotting. The Pph protein eluted in fractions 43-46 (Figure 7B). The molecular weight of the Pph protein complexes was estimated by comparison with standard proteins on the same column. A majority of the Pph protein eluted at about 35 kDa (fraction 45) but a substantial amount was found as dimers at 70 kDa (fraction 43). A higher molecular weight form of Pph was found in fraction 22/23 above the exclusion limit of the column (200 kDa) and contains most likely higher aggregates which were also previously observed with Ppr [36,37]. To verify the oligomeric states, fractions 43-46 were run on a non-reducing SDS-PAGE. Two protein bands with a molecular weight of about 35 and 70 kDa, respectively, were detected and analyzed by MALDI-TOF mass spectroscopy. The analysis clearly identified the Ppr photoreceptor (data not shown).

### The Pph protein expressed in *R. centenaria *is found in a complex with Rc-CheW

**Figure 8:**
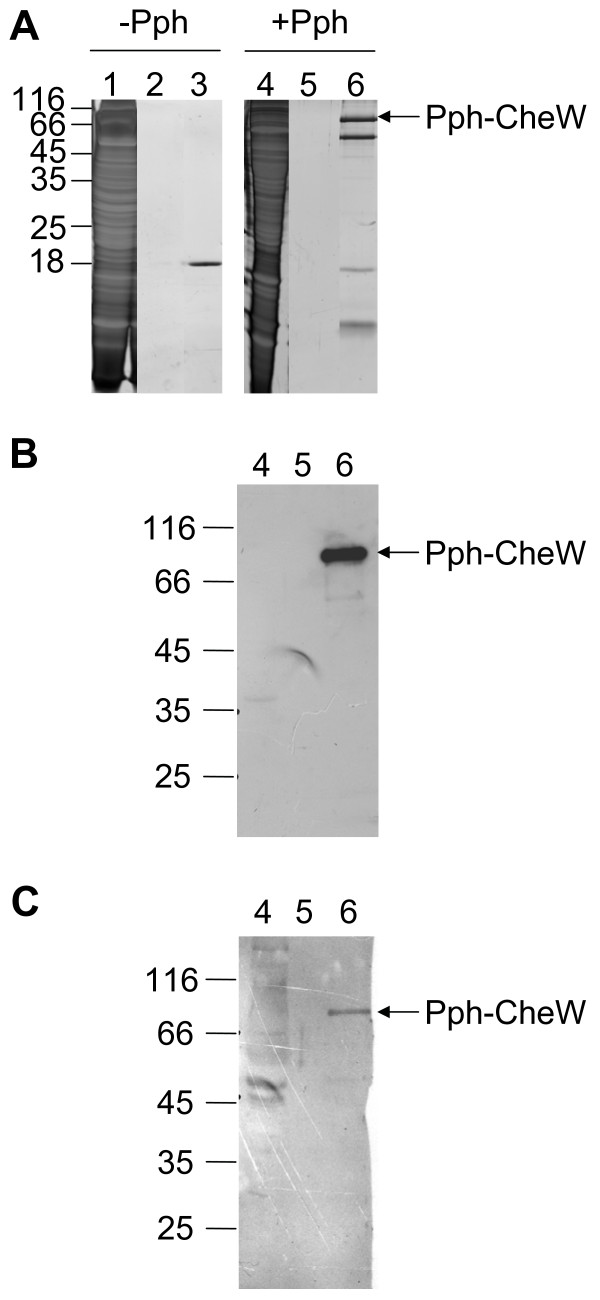
**Protein complexes containing Pph isolated from *R. centenaria*. **The Pph protein C-terminally fused to a strep-tag was expressed in *R. centenaria *and bound to a streptactin-Sepharose column. The elution fractions were analyzed by SDS-PAGE, silver staining (A) and Western blot with antibodies to strep-tag II (B) or to Rc-CheW (C), respectively. The crude protein extract (lanes 1 and 4), the last washing step (lanes 2 and 5) as well as the elution step (lanes 3 and 6) are shown. The positions of molecular weight markers are indicated.

To test whether the Pph protein also assembles into a complex in *R. centenaria *cells, a plasmid containing an oxygen regulated *puc *promoter and an N-terminally his-tagged and C-terminally strep-tagged histidine kinase domain gene was constructed. This plasmid was transferred from *E. coli *RR28 [38] to *R. centenaria *by conjugation and the protein expression was induced by anaerobic growth conditions (see Experimental Procedures). The culture was continued at 42°C for 96 h and the Pph protein was purified using streptactin sepharose. The elution fractions were analyzed by SDS-PAGE, silver staining (Figure 8A) and Western blotting (Figure 8B). At the expected molecular weight of about 35 kDa no monomeric Pph protein was detectable (Figure 8A). In contrast, two bands corresponding to a molecular weight of about 85 kDa and 60 kDa, respectively, were found. The 85 kDa band was recognized by an antibody to the strep-tag epitope (Figure 8B), that is present at the C-terminus of Pph. The 85 kDa band was also recognized by the antibody to Rc-CheW (Figure 8C), suggesting that this band contains a Pph dimer and Rc-CheW protein. The 60 kDa band represents a non-identified protein that bound to the immobilized Pph. In conclusion, a stable complex of Pph and CheW can be isolated from *R. centenaria *cells confirming our *in vitro *findings.

## Discussion

Since photosynthetic bacteria have to locate their habitat with optimal light conditions, specialized sensor systems and signal transduction cascades involving different chromophores arose during evolution (for review see [39]). The blue light sensitive Ppr protein of *R. centenaria *consists of three distinct domains, the Pyp domain containing a cinnamic acid chromophore, the phytochrome-like bilin binding domain and the histidine kinase domain Pph (Figure 1; [22]). The structural organization suggests that the protein is involved in a light-dependent signaling pathway similar to chemotaxis. Since *R. centenaria *exhibits a strikingly obvious phototactic behavior it is compelling to assume that the Ppr protein is involved in this reaction. Light with a wavelength of above 650 nm is attractive, whereas light with less than 650 nm acts as a repellent [10]. The absorption maximum of a prototypical cinnamic acid chromophore in a Pyp light sensor is at about 450 nm [40], whereas the phytochrome-linked biliverdin absorbs red light, suggesting that the latter could function as an attractant sensor. Recently, Cusanovich and co-workers showed that the holo-Ppr of *R. centenaria *has absorption maxima at 425 nm (Pyp), 400, 642 and 701 nm (phytochrome) [36] corresponding to the typical absorption spectrum of Pyp [40] and phytochromes [41]. The phytochromes TaxD1, Cph2 and PlpA were found to be involved in the phototactic reaction of *Synechocystis *sp. PCC 6803, a finding that supports the idea of a participation of the Ppr sensor in the phototactic response of *R. centenaria *[42,43].

The data presented here show that the histidine kinase Pph domain of the Ppr receptor is found in a complex with Rc-CheW when isolated from *R. centenaria *(Figure 8). The interaction of Pph and Rc-CheW was also observed *in vitro *with purified components showing a strong affinity with a K_D _of about 130 nM (Figure 5). In contrast to the trimeric Tsr-CheA-CheW complex that is formed in *E. coli *with an affinity of about 3 μM [16] we observed that the complex formation of Pph and Rc-CheW is clearly ATP-dependent (Figure 4B). It is likely that the Pph-CheW complex is capable to bind Rc-CheAY (Figure 6) consistent with the idea that the chemotactic network is functioning in the presence of Pph. However, the function of the Rc-CheAY fusion protein in this signaling cascade remains unclear. Preliminary transphosphorylation experiments that we perfomed indicate that the CheY domain of the Rc-CheAY protein acts as a phosphate receiver domain and that the CheY domain acts as a phosphate sink similar as it has been described for the chemotactic system in *Rhizobium meliloti *and *Helicobacter pylori *[44,45]. The involvement of Ppr in chemotaxis is also supported from the experiments we performed with *E. coli*. The heterologous expression of Pph has a strong inhibitory effect on chemotaxis as demonstrated by the swarm assay (Figure 2) and the capillary assay (Figure 3). Both assays showed that upon expression of Ppr or Pph the chemotaxis of *E. coli *is turned off whereas expression of the *R. centenaria *histidine kinase KdpE had no effect. This suggests that the Ppr protein interacts with Ec-CheW although the CheW proteins of *E. coli *and *R. centenaria *show a homology of only about 59% and an identity of 28% [12]. However, the structural analysis suggests that all CheW proteins of different species share common features [46,47]. We propose that the binding of the Ppr protein results in a non-functional Ec-CheW-Ppr complex that is inhibitory for chemotaxis (Figures 2 and 3) due to the inactivation of Ec-CheW. Remarkedly, a mutant of the predicted phosphorylation site of Pph with the histidine at position 670 being changed to an alanine residue had a less inhibitory effect on chemotaxis, suggesting that the kinase activity of Pph has a functional role in CheW binding. Similar inhibitory effects on chemotaxis have been observed for *E. coli *when Ec-CheW, Ec-CheA or the MCP-receptors were overproduced [23,25,27]. In addition, such an inhibitory effect was also observed when chemotactic proteins from other organisms like *Rhodobacter capsulatus *[48] or *Leptospira interrogans *[46] were heterologously expressed in *E. coli*.

We found that the histidine kinase domain Pph was mainly present as a monomer when expressed in *E. coli *(Figure 7) and only a minor fraction was found as dimers. Most other bacterial histidine kinases that have been investigated so far were found to be homodimers [49]. Accordingly, when the plasmid encoded Pph protein was isolated from *R. centenaria *it appeared in a complex consisting of CheW and most likely a dimer of Pph (Figure 8).

## Conclusions

Working in a network together with the perception of chemical substrates by the chemotactic signal transduction cascade, Ppr could very well be a part of a complex sensory machinery to tune metabolic and phototrophic processes in phototrophic bacteria. The perception of light may only be an oblique indicator for the metabolic state of a *R. centenaria *cell as is suggested by its influence on cyst formation [13,22]. Therefore, Ppr could work in parallel with the photosynthetic electron transport sensor Ptr of *R. centenaria *[50] to specifically regulate cellular motility and sense the metabolic state of the cell.

## Methods

### Bacterial strains and culture conditions

All genetic manipulations were performed according to standard methods in *E. coli *XL1-Blue (*recA1 thi supE44 endA1 hsdR17 gyrA96 relA1 lac *F′ (*pro*AB^+ ^*lacI*^q ^*lacZ*ΔM15 Tn*10*) as described [51]. For expression of Rc-CheW and Pph, *E. coli *C41 [52] was used. For genetic transfer into *R. centenaria, E. coli *RR28 [38] and in the swarm assays, *E. coli *MM500 [53] was used. For *E. coli*, antibiotics were added at final concentrations of 200 μg/ml ampicillin, 10-50 μg/ml kanamycin and 5 μg/ml gentamycin and for *R. centenaria *5 μg/ml gentamycin, 10 μg/ml kanamycin. All *E. coli *strains were cultured in LB medium at 37°C if not indicated otherwise. *R. centenaria *(ATCC 43720) was obtained from the culture collection. (For anaerobic photosynthetic growth *R. centenaria *was cultured in screw cap bottles filled to the top with PYVS medium [10] and illuminated by an 80 W tungsten bulb (Concentra, Osram, Germany) at 42°C.

### Construction of Pph and Che Plasmids

The plasmids used in this study are described in Table 1. The gene fragment coding for the histidine kinase domain Pph was amplified by PCR using the cloned *ppr *gene in pT-Adv as a template (Clontech). The *Nde*I and *Nsi*I restriction sites were introduced with the primers PYP-Nde (5'-CAGCGGCATATGCCGCGCATCTCCTT-3') and PYP-Nsi (5'-GATCAGGCCCCGATATGCATGGTGACGGT-3'). The resulting ~0.9 kb fragment was ligated and subcloned in pT7-7 [54] using *Nde*I and *Eco*RI. A spacer sequence (5'-CAGCCGGGCGGTGCAGGCTCAGGCATG-3') and the StrepTag II oligonucleotide (ATCCAACTGGTCCCACCCGCAGTTCGAAAAAATGC-3') were inserted into the *Nsi*I-site to give plasmid pSK4. To generate pET16b-Pph the pSK4 plasmid was cut by *Nde*I and *Bam*HI and the corresponding ~0.9 kb fragment was ligated into the pET16b vector (Novagen). Construction of plasmid pBAD-Pph was performed as follows. pET16b-Pph was digested by *Xba*I and *Hind*III and the resulting fragment was inserted into the corresponding restriction sites of pBAD18 [55]. All genetic manipulations were verified by DNA-sequencing.

**Table 1:** Constructs and plasmids used in this study

Plasmids	Characteristics	Reference
NB10	Amp^r^, pET16b, His_10_-Ppr	[63]
pBAD18	Amp^r^, *araC*, P_BAD_, pBR322 *ori*, expression vector	[55]
pBAD-Pph	Amp^r^, pBAD18, His_10_-Ppr_Δ1-601_-StrepTag II	this study
pBAD-PphH670A	Amp^r^, pBAD18, His_10_-Ppr_Δ1-601; H670A_-StrepTag II	this study
pBAD-Ppr	Amp^r^, pBAD18, His_10_-Ppr-StrepTag II	this study
pBAD-KdpE	Amp^r^, pBAD24, Rc-KdpE	this study
pET16b	Amp^r^, T7 promoter, N-terminal deca histidyl tag	Novagen
pET16b-Pph	Amp^r^, pET16b, His_10_-Ppr_Δ1-601_-StrepTag II	this study
pET16b-PphH670A	Amp^r^, pET16b, His_10_-Ppr_Δ1-601; H670A_-StrepTag II	this study
pET16b-Ppr	Amp^r^, pET16b, His_10_-Ppr-StrepTag II	this study
pET28-CheAY	Kan^r^, pET28a(+), His_6_-CheAY	[12]
pRK2013	Kan^r^, Tra, helper plasmid	[58]
pSK10	Gen^r^, pZJD11	this study
pSK4	Amp^r^, pT7-7, Ppr_Δ1-601_-StrepTag	this study
pT7-7	Amp^r^, T7 promoter, expression vector	[54]
pT7-7-CheW	Amp^r^, pT7-7, His_6_-Rc-CheW	this study
pT-Adv	Amp^r^, Kan^r^, lac promoter, *lacZ*	Clontech Inc.
pZJD11	Gen^r^, pRK2 derived plasmid, *lacZ*	[12]

A *ppr-strep tag II *fusion gene was constructed as follows. pET16b containing the entire *ppr *gene (pNB10), as well as pET16b-Pph were cut by *Nco*I and the resulting fragments (~6.0 kb and ~2.5 kb) were ligated. The orientation of the *ppr*-insert was checked by DNA-sequencing and the resulting plasmid was named pET16b-Ppr. To construct an arabinose inducible full length *ppr*, the gene was excised by *Xba*I and *Hin*dIII from pET16b-Ppr and ligated into the pBAD18 vector.

The putative phosphorylation site (the histidine at position 670 in the Ppr protein) was changed to an alanine (CAC→GCG) using site directed mutagenesis with the primers (5'-CTGGCGAACATGAGCGCGGAGCTGCGGACTCCG-3') and (5'-CGGAGTCCGCAGCTCCGCGCTCATGTTCGCCAG-3') and pSK4 as a template. The resulting mutant was digested by *Nde*I and *Bam*HI and subcloned into the pET16b vector generating pET16b-PphH670A. Then the *pph*H670A mutant was excised by *Xba*I and *Hind*III and the fragment was inserted into the pBAD18 vector to create pBAD-PphH670A.

To express the histidine kinase domain Pph with an N-terminal his_10_-tag and a C-terminal strep-tag II in *R. centenaria*, the plasmid pZJD11 (kindly provided by C. Bauer) was used [12]. We used the oxygen regulated *puc *promoter and the *puh*A Shine Dalgarno sequence from *Rhodobacter capsulatus *to initiate translation. Therefore, a PCR reaction with the primers (5'-TACGTAGGGCCCTAAGCTAAAGGAGGACTAACATGGGCCATCATCAT-3') and (5'-TACGTAGGCGCGAATTCGGCTTGATCAGGC-3') and pET16b-Pph as a template was conducted. Simultaneously, a *Sna*BI restriction site was introduced at the 3' end of the gene. The resulting fragment was subcloned into pGEM T-easy vector (Promega) and verified by DNA sequencing. This plasmid was used as a template to insert the *puc *promoter via a second PCR. The primers (5'-GGTAACCTTGATCGCCGACACTTGGGCTCCCA TAGTGGAGCTCGGGCCCTAAG-3') and (5'-TACGTAGGCGCGAATTCGGCTTGATCA GGC-3') were used to introduce a *Bst*EII site at the 5' end. The resulting fragment was inserted into pGEM T-easy vector. After sequencing, the *pph *construct was excised by *Bst*EII and *Sna*BI and ligated into the corresponding sites of pZJD11 to generate pSK10.

To express the Rc-CheW protein in *E. coli*, the *cheW *gene was amplified by PCR from the *R. centenaria *genome using the primers (5'CATATGCATGCCCGCCTGCCCGTTCCC-3') and (5'GGGAATCGTTCATTGCGATCAGTTTCCGG-3'), respectively. The resulting fragment was first cloned into pT-Adv. Then the *cheW *gene was excised by *Nde*I and *Eco*RI and ligated into the corresponding restriction sites of a pT7-7 derivative containing a decahistidine sequence to create the IPTG inducible expression vector pT7-7-CheW.

### Swarm agar assays

TB swarm agar plates (1% bacto-tryptone, 0.8% NaCl; 0.35% bacto-agar) containing 0.2% arabinose or 0.2% fructose, respectively, were inoculated with a single colony of *E. coli *MM500 or MM500 harbouring one of the plasmids pBAD-Ppr, pBAD-Pph, pBAD-PphH670A, pBADKdpE and pBAD, respectively. The plates were incubated for 6 hours at 37°C.

### Chemotaxis assay using a chemotactic chamber

2 ml minimal medium A (MMA) [56] containing an amino acid mixture (threonine, leucine, histidine, methionine), vitamin B1 (final concentration 10 μg/ml each), 200 μg/ml ampicillin and 0.2% fructose were inoculated with an overnight culture of *E. coli *MM500 or cells harbouring pBAD-Pph, pBAD-PphH670A, pBAD-KdpE or pBAD18, respectively. When the cultures reached an OD_600 _= 0.6 the cells were washed twice with MMA without sugar and finally either 0.2% arabinose to induce protein expression or 0.2% fructose (as a control) were added. The cultures were incubated for 60 min at 37°C. For the kinetic analysis the incubation times are indicated in Figure 3B. Again, the cells were washed twice with MMA without carbon source and were back diluted to an OD_600 _= 0.6. The chemotactic assays were performed as follows. 300 μl of the cell suspension were filled in each drilling of the chamber and a capillary containing either 2 μl 1 mM aspartate or 2 μl H_2_O as a control was placed into the channel between the two cylindrical compartments. The chamber was incubated at 37°C for 30 minutes. The outside of the capillary was washed extensively with sterile water and the content of the capillary was blown out and a dilution series was streaked on agar plates. After overnight incubation at 37°C the colonies were counted and the chemotactic inhibition (CI) was calculated as the ratio of colonies of the water containing capillary to the colonies from the aspartate containing capillary. Therefore, a low CI indicates an undisturbed chemotactic response whereas a high CI reflects an inhibition of the *E. coli *chemotactic system.

### Expression and purification of Pph protein from inclusion bodies

*E. coli *strain C41 [52] harbouring the plasmid pET16b-Pph were grown at 37°C in 1 l LB medium containing 200 μg/ml ampicillin. When cells reached the midlogarithmic phase, IPTG was added at a final concentration of 1 mM and the cells were grown for an additional 4 hours at 37°C. Then the cells were harvested by centrifugation. The resulting pellets were resuspended in 100 mM Tris-HCl pH 8.0, 150 mM NaCl (buffer W) and lysed by a French Press. Inclusion bodies were precipitated by centrifugation and resuspended in buffer W containing 0.5% N-lauroylsarcosine. The inclusion bodies were solubilized overnight at 4°C with gentle shaking. To the filtered extract 10 mM imidazole was added and applied to a Sepharose 6b (GE Healthcare) column precharged with Cu(II) ions. Unbound proteins were removed by washing the column with 15 column volumes of buffer W containing 0.5% N-lauroylsarcosine and 10 mM imidazole. The bound protein was eluted by a linear gradient up to 500 mM imidazole in buffer W + 0.5% N-lauroylsarcosine. The Pph protein containing fractions were pooled, diluted 1:40 with buffer W (final detergent concentration = 0.01%) and applied to a streptactin-sepharose column (IBA, Göttingen, Germany) to remove contaminating proteins. After washing the column with five column volumes buffer W + 0.01% N-lauroylsarcosine, the protein was eluted with buffer W + 0.01% N-lauroylsarcosine containing 2.5 mM desthiobiotin. The protein was dialyzed against buffer W + 0.01% N-lauroylsarcosine and the purity was checked by SDS-PAGE analysis as described [57]. Protein marker SM0431 and SM0441(Fermentas) were used.

### Expression and purification of Rc-CheW

1 Liter of LB medium containing 200 μg/ml ampicillin was inoculated with a freshly transformed single colony of *E. coli *C41 harbouring the plasmid pT7-7-CheW. The cells were grown to a cell density of 2 × 10^8 ^cells per ml at 37°C, then IPTG was added to a final concentration of 1 mM. The cells were incubated for an additional 4 hours and harvested by centrifugation. The pellet was resuspended in TBS (50 mM Tris-HCl pH 7.4, 150 mM NaCl) and lysed by a French Press. Cell debris was removed by centrifugation and a final concentration of 10 mM imidazole was added. This crude extract was applied to a Cu(II)-charged Sepharose 6b column and unbound proteins were washed out with 10 column volumes of TBS + 10 mM imidazole. The protein was eluted with a linear gradient from 10 to 500 mM imidazole and fractions containing Rc-CheW were dialyzed against TBS-buffer. The homogeneity of the protein was monitored by SDS-PAGE.

### Expression of the Pph protein in *R. centenaria*

The plasmid pSK10 was transferred to wild type *R. centenaria *by triparental conjugation using *E. coli *RR28 [38], the helper plasmid pRK2013 [58] and the filter-mating technique as described previously [59]. After conjugation, about 10^9 ^T7 phages were added, and the mixture was incubated for 30 minutes at 37°C to eliminate remaining *E. coli *cells. Finally, conjugants were selected on the basis of gentamycin resistance on PYVS plates containing 5 μg/ml gentamycin under anaerobic conditions. 2L PYVS media containing 5 μg/ml gentamycin and 10 μg/ml kanamycin (*R. centenaria *is naturally resistant to kanamycin [12]) was inoculated with a culture of pSK10 containing *R. centenaria *cells. The cells were grown under anaerobic and illuminated conditions for 96 h and harvested by centrifugation, resuspended in 100 mM Tris pH 8.0, 150 mM NaCl (buffer W) and lysed by a French Press. The cell debris and the photosynthetic membranes were removed by centrifugation. The cleared extract was applied to a streptactin-sepharose column (IBA). The unbound proteins were removed by extensively washing the column with buffer W and the bound proteins were eluted with buffer W containing 2.5 mM desthiobiotin. The success of the purification was verified by SDS-PAGE, silver staining and Western blot analysis with the antibodies raised against the his-tag or the strep-tagII, respectively.

### Determination of the dissociation constant of Pph and Rc-CheW by resonant mirror spectroscopy

The Pph protein was purified from inclusion bodies as described above and the aminosilane cuvette was activated as described by the manufacturer (Iasys, Biosensors). 200 μl of the purified Pph protein (50 μg/ml) was added to the activated cuvette and the immobilization was recorded for 30 minutes. The unbound protein was removed by extensive washing and increasing amounts of purified Rc-CheW (see above) were added. After 30 minutes of incubation the free Rc-CheW was washed out and the amount of bound Rc-CheW was determined for each experiment. The fractional saturation was $\bar{p}$ calculated and depicted against the amount of the added Rc-CheW concentration. The resulting Scatchard Plot is illustrated as the inlet of Figure 5.

### *In vitro *transcription and translation

The histidine kinase domain Pph as well as Rc-CheAY were transcribed *in vitro *from the plasmids pSK4 and pET28-CheAY, respectively, using a T7 transcription kit (Fermentas) according to the manufacturers manual. The translation reaction was performed as described previously [60] by using an *E. coli *based cell free expression system The proteins were labeled with 10 μCi of [^35^S]methionine (ICN) in each experiment. The high speed supernatant (S-135) was prepared as described from *E. coli *MRE600 [61].

### Pull-down assays

50 μg of the purified his_6_-Rc-CheW protein was mixed with 25 μl of the *in vitro *translated Pph protein and 25 μl Rc-CheAY when indicated. The protein mixture was incubated overnight at 37°C. Then, the his_6_-Rc-CheW protein was bound to a column containing 50 μl Sepharose 6b (GE Healthcare) charged with Cu(II) ions and pre-equilibrated with buffer I (20 mM sodium phosphate pH 7.7, 200 mM NaCl, 50 mM imidazole pH 8.0). After 30 minutes at room temperature, the unbound proteins were removed by washing the column five times with 500 μl buffer I followed by an elution with 1.5 ml buffer II (20 mM sodium phosphate pH 7.7, 200 mM NaCl, 500 mM imidazole pH 8.0). All fractions were TCA precipitated and analyzed by SDS-PAGE. The gels were stained with coomassie brilliant blue and the radiolabeled bands were quantified using a Fuji BAS 1500 phosphorimager.

### Gelfiltration assay

1L terrific broth [62] in a Fernbach flask was inoculated with an overnight culture of *E. coli *C41 (DE3) harbouring pET16b-Pph. The cells were incubated at 18°C with gentle shaking for 48 hours. This procedure prevents the formation of inclusion bodies [36]. Then the cells were harvested by centrifugation and resuspended in 20 mM Tris pH 7.4, 40 mM NaCl, 20% glycerol. The cells were lysed by 3 passages through a French Press and the cell debris was removed by centrifugation. The filtered crude protein extract was applied on a Sephadex G-200 gelfiltration column (GE Healthcare) and separated according to the manufacturer's manual. The resulting fractions were analyzed by SDS-PAGE and Western blotting with an antibody to strep tag II (IBA, Göttingen, Germany).

## Authors' contributions

SK and DK performed the experiments, AK and DK designed and coordinated the project. All authors contributed in the writing of the manuscript and approved the final manuscript.
