# Erratum to: The photosensor protein Ppr of *Rhodocista centenaria* is linked to the chemotaxis signalling pathway

**DOI:** 10.1186/s12866-017-1071-x

**Published:** 2017-08-23

**Authors:** Sven Kreutel, Andreas Kuhn, Dorothee Kiefer

**Affiliations:** Institute of Microbiology and Molecular Biology, Garbenstrasse 30, University of Hohenheim, 70599 Stuttgart, Germany

## Erratum

After publication of our article [1] we became aware that two errors had been introduced during the revision process. These errors affect two figures (Fig. 2 and Fig. 4):

- In Fig. 2B the control panels with the non-transformed cells were wrong.
- In Fig. 4A the right panel (+CheW) was wrong.

**Fig. 2:**
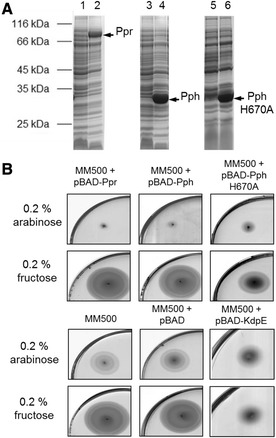
Chemotaxis of *E. coli* is inhibited by the expression of Ppr or Pph. **a** The chemotactic wild type strain *E. coli* MM500 was transformed with the plasmids pBAD-Ppr (lanes 1 and 2), pBAD-Pph (lanes 3 and 4) and pBAD-Pph H670A (lanes 5 and 6). Cells were grown in TB medium to an OD_600_ = 0.5, 0.2% fructose (lanes 1, 3 and 5) or 0.2% arabinose (lanes 2, 4 and 6) was added, and growth was continued for 3 h. Protein expression was analyzed by SDS-PAGE and Coomassie blue staining. The positions of molecular weight markers are indicated. **b** TB swarm agar plates containing either 0.2% arabinose (*upper* panels) or 0.2% fructose (*lower* panels) were inoculated with *E. coli* MM500 cells bearing the plasmids pBAD-Ppr, pBAD-Pph and pBAD-Pph H670A, pBAD (vector without insert) or pBAD-KdpE. To develop chemotactic rings the plates were incubated for 6 h at 37 °C

**Fig. 4:**
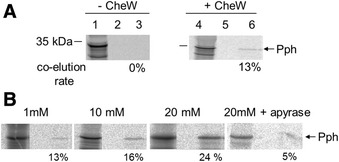
Interaction between Pph and the chemotactic protein Rc-CheW. **a** The binding of the histidine kinase domain Pph and CheW was analyzed in pull-down assays. *R. centenaria* 6his-Rc-CheW was expressed in *E. coli* C41 cells and purified. The Pph protein was translated in vitro in the presence of [^35^S]-methionine (lane 1 and 4). Rc-CheW was added (50 μg) to the reaction and incubated at 37 °C. The sample was applied to a Cu-Sepharose column and after washing the bound complexes were eluted (lanes 3 and 6). The fractions were analysed by phosphorimaging. The in vitro translating protein extracts are shown in lanes 1 and 4, the final wash steps in lanes 2 and 5 and the elution fractions in lanes 3 and 6, respectively. The co-elution rate was calculated and is indicated. The positions of molecular weight markers are indicated. **b** The binding of the Pph protein and Rc-CheW was analysed in the presence of ATP. The Pph protein was translated and Rc-CheW was added as described in (A). ATP or apyrase was added to each reaction as indicated and the samples were analysed as described in (A). The co-elution rate was calculated and is indicated in % as bound Pph protein

Neither error changes the outcome of the experiments or the conclusions of the article. The corrected figures are shown as follows:
